# A meta-barcoding analysis of soil mycobiota of the upper Andean Colombian agro-environment

**DOI:** 10.1038/s41598-019-46485-1

**Published:** 2019-07-12

**Authors:** Angela Landinez-Torres, Simona Panelli, Anna Maria Picco, Francesco Comandatore, Solveig Tosi, Enrica Capelli

**Affiliations:** 1 Mycology Laboratory, Department of Earth and Environmental Sciences, University of Pavia, Via Ferrata 7, 27100 Pavia, Italy; 2 Facultad de Ciencias Agrarias y Ambientales, Fundación Universitaria Juan de Castellanos, Carrera 11 No. 11 - 44, Tunja, Boyacá Colombia; 3 Pediatric Clinical Research Center Romeo ed Enrica Invernizzi, University of Milan, Via G.B. Grassi 74, 20157 Milan, Italy; 4 Genetic Analysis Laboratory, Department of Earth and Environmental Sciences, University of Pavia, Via Ferrata 7, 27100 Pavia, Italy; 5 Centre for Health Technologies (C.H.T.), University of Pavia, Via Ferrata 1, 27100 Pavia, Italy

**Keywords:** Biological sciences, Biological sciences, Biodiversity, Fungal ecology, Fungal ecology

## Abstract

Colombia is a country for which one of the highest biodiversity rates is reported, and one of the first in the tropical areas where an effort was made to gather information on indigenous fungi. Nevertheless, mycological data are still scarce and discontinuous, above all on soil fungi. The present study wanted to contribute to unveil the large soil fungal biodiversity in the upper Andean Colombian agro-ecosystems. The studied area is located in the department of Boyacà, considered with a notable economical value, partly devoted to subsistence agriculture. More than 150 described species were revealed in this study, belonging to 5 phyla with *Ascomycota* representing the dominant taxon. *Basidiomycota* and *Zygomycota* are also well represented, dominated by species of the genus *Sebacina* and *Mortierella* respectively, mainly distributed in the semi-natural plots (woodland and grassland). Most of the species are reported as first records for Colombia. Some of them are particularly interesting for their conservation significance such as *Geoglossum fallax*, which is the dominant species in the unimproved grassland plot. The bootstrap-based clustering analysis showed a different distribution of the species in orchards and non-cultivated areas as a possible response of the fungal community to different use of soil in the agro-environment.

## Introduction

The assessment of the global diversity of soil fungi is often demanding due to the microscopic dimension of many species and to the fact that most of them are hidden^1^. In the *Dictionary of Fungi*^2^, the entry “number of Fungi” results in a total of 97,330 species. This number raises to about 99,000 with the addition of the 1300 Microsporidia species listed by Hibbett *et al*.^3^. Even with this, the gap with the estimated number of species ‒ 1.5–5.1 million^4^ ‒ remains relevant^5^. However, in the last two decades culture-independent methods based on sequence-specific identification have deeply reshaped research in the field, especially with reference to uncultivable species, becoming invaluable tools for their ability to effectively lead to new species discovery in a shorter time^6,7^. This applies also to soil, one of the most complex habitats and richest reservoir of bacteria and fungi of the biosphere. Our knowledge on the fungal biodiversity in soil has been continuously increasing, largely thanks to the cited molecular techniques^8,9^, comprised, in the last years, those based on metagenomics, metabarcoding and next-generation sequencing platforms^10^. For example, in a recent paper Tedersoo *et al*.^11^ analyzed 365 soil samples from natural locations all over the world, using pyrosequencing, to correlate fungal biodiversity to the most various environmental factors. Their data depicted a soil fungal biodiversity highly influenced by mean annual precipitations and distance from equator. The highest fungal diversity, as well as the highest rates of endemism were found to be strongly linked to the tropical band^11,12^.

The main contributions of soil mycobiota to the functioning of ecosystems are linked to soil stabilization and nutrient cycling, and thus to the concept of soil quality^13^. In the particular case of agricultural soils, unraveling the composition of their mycobiota provides the basis for better understanding functions exerted by the microbial component (fungi and bacteria), also in view of a more effective managing of the agroecosystems themselves. Metagenomics and metabarcoding-based analyses of myco-microbiota of various agricultural soils have been faced by several authors, in South America^14–16^, Europe^17–19^ and other locations^20^. Data are consistent with the conclusion that agricultural activities, together with other external stimuli, influence taxonomic composition as well as the metabolic activities of microbial communities. Metagenomic characterization, on the other hand, has allowed to address the influence of agricultural interventions on the soil ecosystem and potentially represents precious tools for defining plant protection strategies^21^.

The present work focuses on the mycobiota of Colombian soils by a metabarcoding analysis, in the furrow of the work recently initiated by Alvarez-Yela *et al*.^16^ that analyzed the fungal phyla inhabiting “paramos” ecosystems of the Andean mountains.

Colombia is one of the countries for which the highest biodiversity rates are reported. 35,000 plant species have been recorded and, according to the association between plants and fungi proposed by Hawksworth^22^, it can be assumed that, in Colombia, fungal species must be >210,000. Even if this country was one of the first in the tropical area where an effort was made to gather information on the mycological flora, also in association to agroecosystems, data still appear scarce and discontinuous. The work of Fuhrmann and Mayor^23^ on Colombian parasitic fungi dates back to 1914 and the publication of Chardon and Toro^24^ was the first which referred comparative results of large explored areas as reported by Dumont *et al*.^25^. Gradually, the characterization of Colombian fungal species was enriched thanks to serial publications on the *New or noteworthy fungi from Panama and Colombia*, mainly coming from the Sierra Nevada of Santa Marta, in the department of Magdalena^26–29^. In 1974, the cooperative program *Mycological Flora of Colombia* was launched, and more than 2,000 fungi in the departments of Cundinamarca, Antioquia, Valledel Cauca and Boyacá were recorded^25^.

The attention to microfungi has increased more recently, but knowledge on their diversity and ecology is still to its infancy^30–39^.

**Figure 1:**
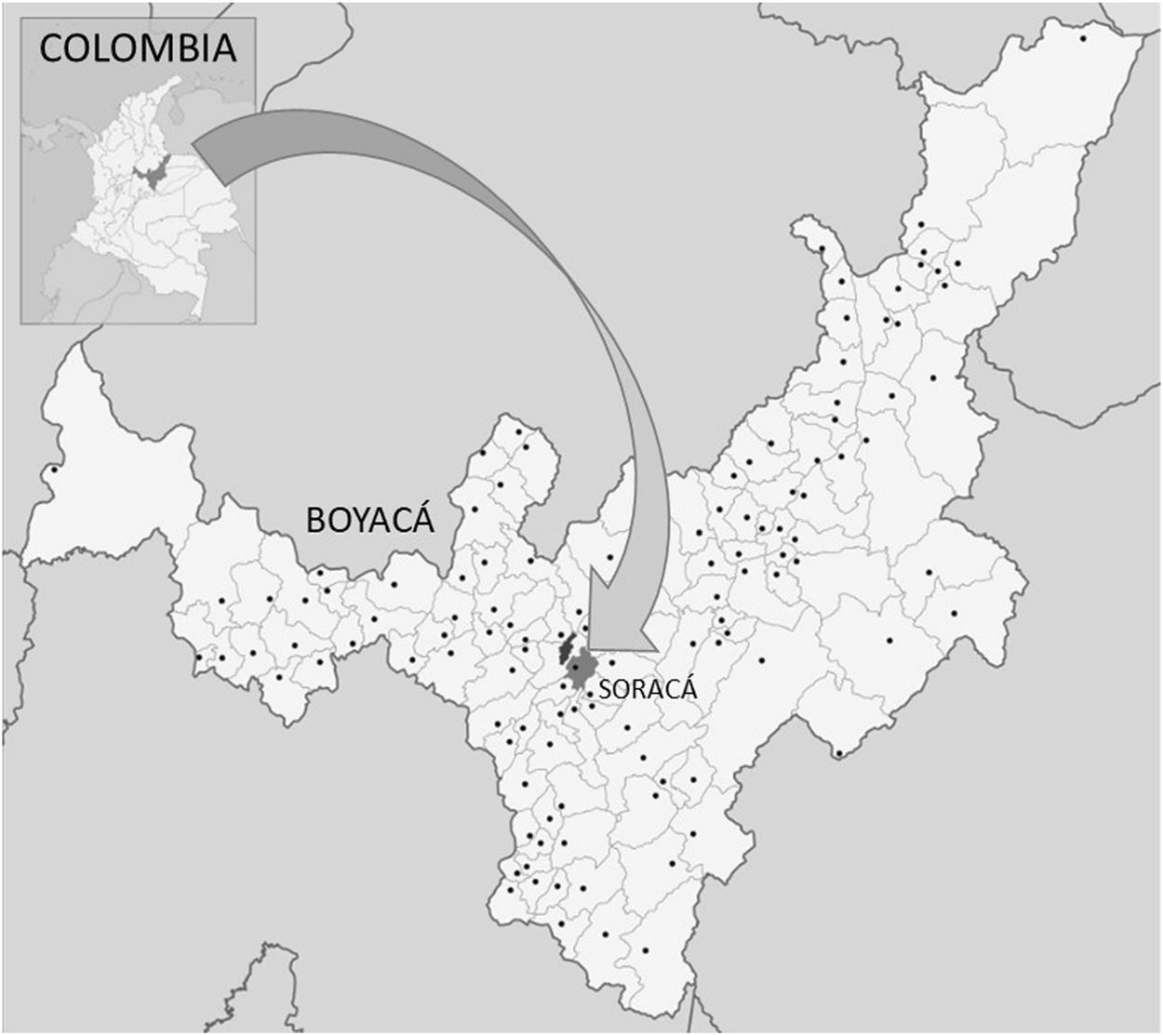
Study area. Department of Boyacá, Colombia, South America. Adapted from Shadowxfox^66^.

The present research wants to contribute to uncover the soil fungal diversity of the upper Colombian Andean agro-environment of the Boyacá department (Fig. 1).

The study area is located on a high plateau (2,800–3,200 m-asl) (Cundiboyacense region) in the Eastern Cordillera of the Colombian Andes.

This area belongs to Colombian paramos, covers part of Cundinamarca and Boyacá departments and belongs to the altitudinal vegetation belt of the upper Andean forest (upper mountain cloud forest). Since approximately 50 years, human activities have transformed the native forest into a grassland, mostly devoted to subsistence agriculture (potatoes and onions). On the other hand, this area is now knowing important changes and becoming a potential flywheel of socio-economic rescue. Since 2012, in the context of improving the economic picture, some scientific and economic projects aim to introducing economically relevant cultures, such as fine varieties of peaches and apples. In view of this, an important piece in the puzzle of the conceivable forthcoming evolution is, for sure, to acquire a deep knowledge of the microbiological condition of this land, to guide choices aimed at its sustainable use and management.

## Results and Discussion

### Physicochemical analysis of soil

**Figure 2:**
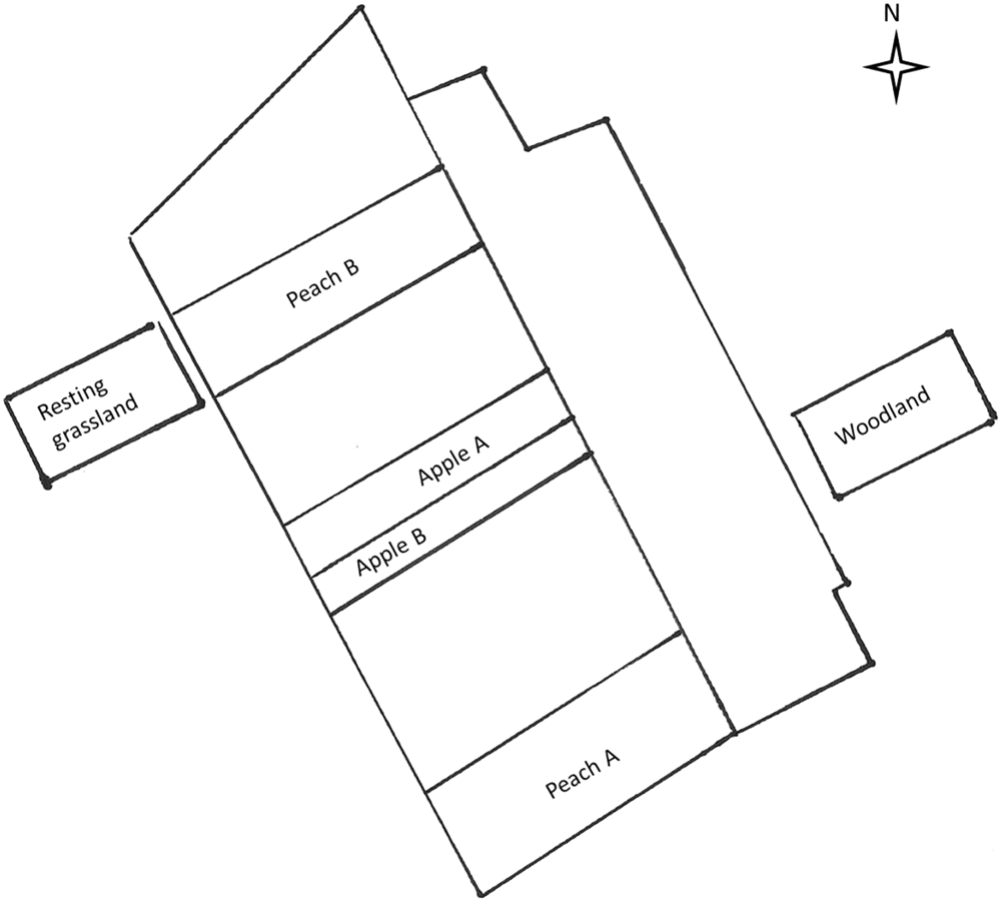
Map of the experimental farm San Isidro Labrador belonging to the Fundación Universitaria Juan de Castellanos, located in the Village of Soracá (Tunja, Colombia). The sampling sites are reported.

Table 1 reports the results of the physico-chemical analyses of the soil samples, collected in the study area (Fig. 2). Overall, the studied samples appear similar in their soil characteristics except for the P concentration detected in the apple A plot, that was the only one with an extremely high value (400 mg/kg), while the other samples were characterized by low (<7 mg/kg) to rich levels (>45 mg/kg).

**Table 1:** Phsyco-chemical characteristics of the sampling sites.

Plot	pH ± 0.10	Organic matter (%)	Ntot (g/kgSS)	Corg (%SS)	C/N	P (mg/kg SS)	Ca (meq/100 g)	Mg (meq/100 g)	K (meq/100 g)	Sand (%)	Silt (%)	Clay (%)
Peach-A	6.6	4.9	2.9	2.89	1	3.6	23.4	5.83	5.49	37.5	41.5	16.2
Peach-B	6	4.22	2.71	2.57	0.9	52	13.5	3.37	4.6	33.2	44.4	17.9
Apple-A	6	5.14	3.2	3.26	1	400	11.5	3.4	5.14	25.8	46.4	19.6
Apple-B	6	4.63	1	2.98	3	9.22	10.9	2	5.86	38.8	38.5	13
Woodland	6	5.43	1.85	3.32	1.8	6.97	9.02	4.35	1.3	34	45.8	14.8
Resting grassland	6.4	4.9	2.12	3.02	1.4	29.6	11.5	4.26	1.75	38	39.9	16.5

### Total count of cultivable fungi

Quantitative mycological analyses have been performed to provide a picture of the total count of cultivable fungi in the analyzed soils (Table 2). Colony forming Units (CFU)/g of soil (dry weight) range from 2 (in resting grassland) to 8 × 10^5^ (in Peach B plot). Differences recorded for CFU values are difficult to be explained by soil physico-chemical data of soil samples which are similar, as already evidenced above; whereas the low value of CFU observed in the resting grassland could be explained by a shortage of water; on the contrary the orchards are irrigated artificially (see Supplementary Table 1) and the woodland is subjected by a less evapotranspiration due to the forest cover.

**Table 2:** Cultivable fungal counts of each study areas. Counts are expressed as colony forming unit per g of soil dry weight.

	Plots					
	appleA	appleB	peachA	peachB	Resting grassland	Woodland
CFU/g of soil dry weight	7 × 10^5^	6 × 10^5^	5 × 10^5^	8 × 10^5^	2 × 10^5^	5 × 10^5^

### Taxonomic picture of fungal communities: phyla, orders, families inhabiting orchards and non-intervened fields

The taxonomic composition of soil fungal communities inhabiting cultivated and non-intervened sites (Fig. 2) was evaluated by deep sequencing of the ITS1 barcode, that produced a total of 856,508 raw reads on the Illumina MiSeq platform, 468,163 after quality filtering. Redundant sequences were removed, and ITS1 reads extracted by the ITSx software. The UNITE ITS reference dataset was finally used for determining operational taxonomic units (OTU) at the 97% level. 25,519 OTUs were returned by the PIPITS wrapper^40^, after removing chimeras. Of these, the amount of unassigned and unidentified OTUs was 0.1% and 8% respectively.

**Figure 3:**
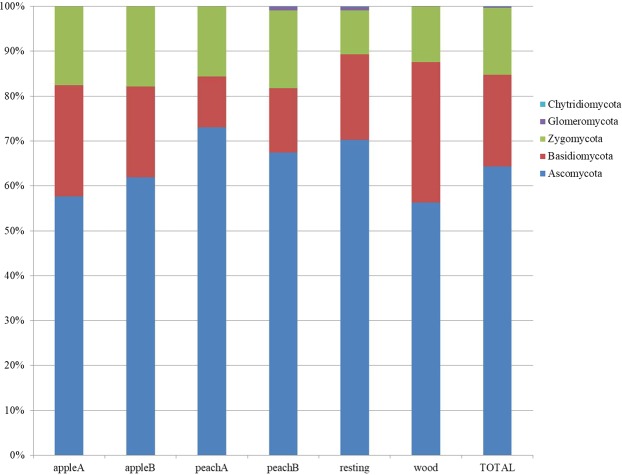
Soil fungal relative abundances at the phylum rank, in the orchards (apple A and B, peach A and B), and controls (resting grassland and woodland soils). The last column shows the average of total abundance in the sampling area.

Taxonomic analyses assigned sequences to five fungal phyla (Fig. 3). As expectable, all areas appear dominated by *Ascomycota*, that accounts on average for the 55% of the total fungal diversity in the plots (Fig. 3, last column), ranging from a minimum of 48% (apple A) to 62% (peach A). *Basidiomycota* represents the 20% ranging from 10% (peach A) to 29% of the woodland sample. *Zygomycota* represent the 15%, with a range from 9% (resting grassland) to 13–14% (cultivated areas). Least, *Glomeromycota* and *Chytridiomycota* sequences have been mostly recorded in the resting grassland, at low frequencies (<0.7% and 0.1% respectively).

**Figure 4:**
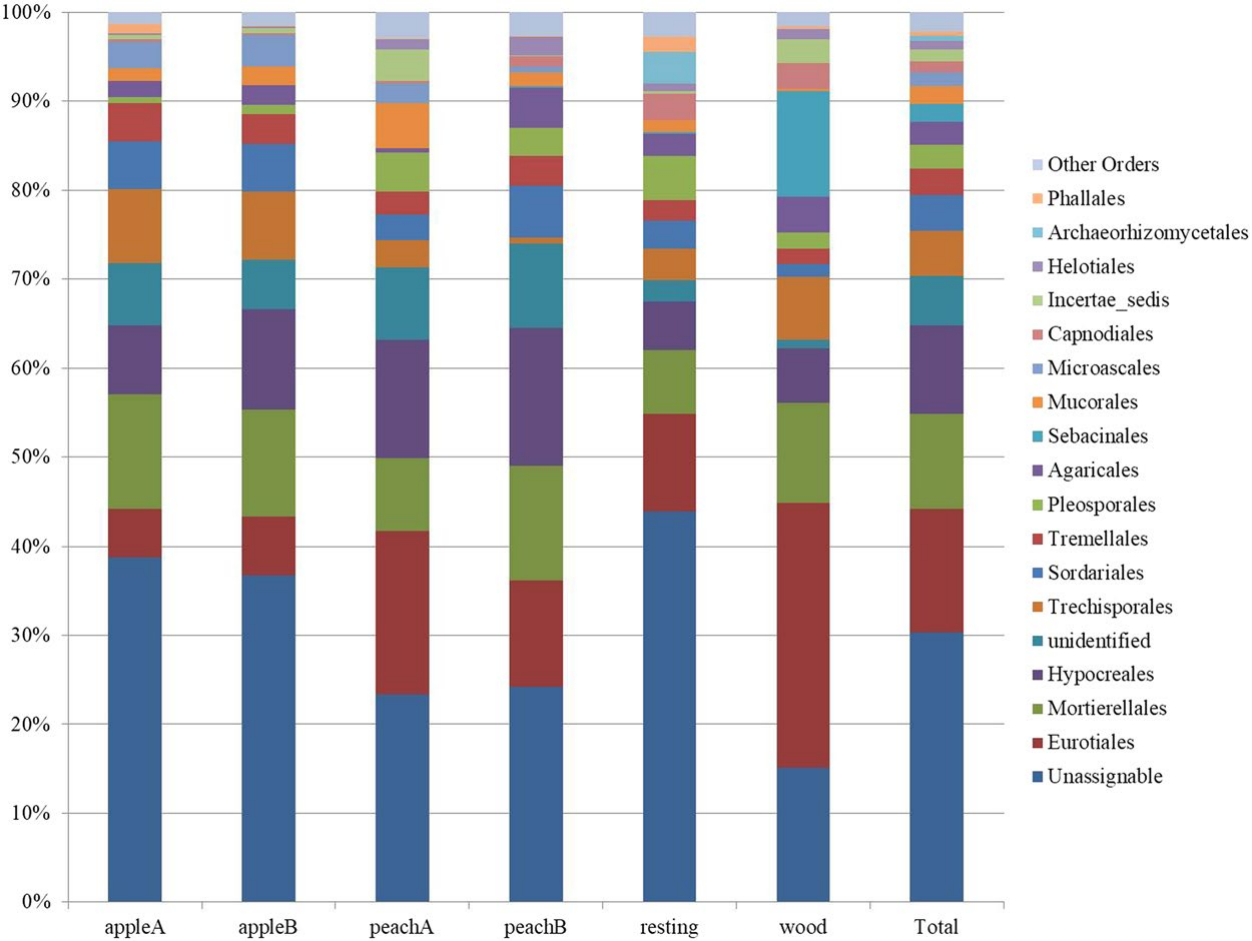
Soil fungal relative abundances at the order level, in the orchards (apple A and B, peach A and B), and controls (resting grassland and woodland soils). The last column shows the average of total abundance in the sampling area.

Figure 4 shows the distribution of fungal orders in the samples, with average values shown in the last column. *Eurotiales* (*Ascomycota*) results the most represented order (20%), followed by *Mortierellales* (*Zygomycota*) (about 15%), and by three orders always belonging to *Ascomycota*: *Hypocreales* (ca 13%), *Trichosporales* (ca 7%), and* Sordariales* (ca 5%). Other orders resulted less represented, with values <5%.

**Figure 5:**
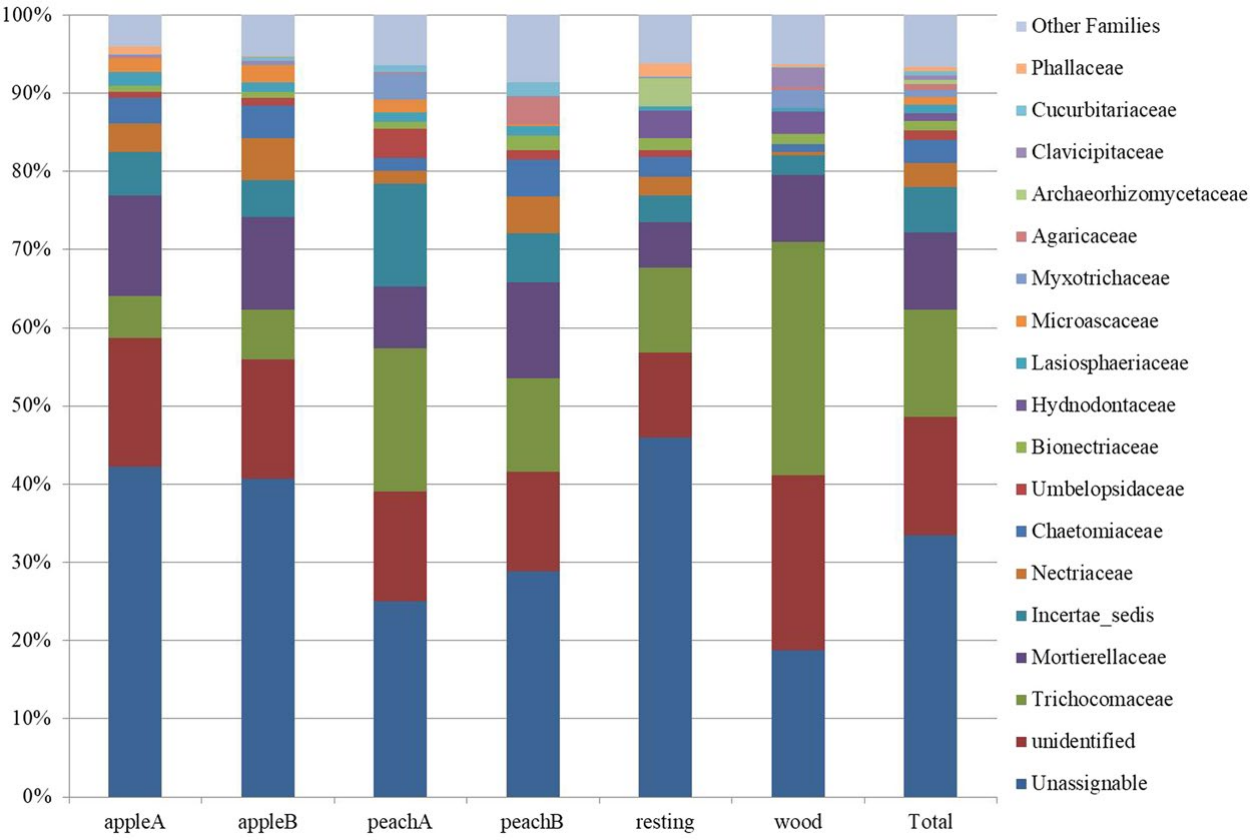
Soil fungal relative abundances at the family level, in the orchards (apple A and B, peach A and B), and controls (resting grassland and woodland soils). The last column shows the average of total abundance in the sampling area.

In Fig. 5 the distribution of the most represented families (frequency ≥1%) is reported. On the whole, *Trichocomaceae* (*Ascomycota*, *Eurotiales*) results the most represented (14%), even if with remarkable differences: the lowest prevalence is retrieved in the apple orchard (5–6%) and the highest (30%) in the woodland soil. *Mortierellaceae* (*Zygomycota*, *Mortierellales*) is the second most abundant family, representing on average 10% of fungal families in the whole sampling area with an opposite behavior with respect to *Trichocomaceae*. Indeed, the lowest prevalence (6%) is here observed in an uncultivated soil (grassland) and the highest in the two orchards: apple A and peach B. It is noteworthy that these are the soils in which the phosphorous concentration was higher (Table 2). The two findings are likely linked due to the known activities of this family, composed by coprophilous genera that respond to organic fertilization and are among the main management-sensitive taxa^17,19^. Finally, *Netriaceae* (*Hypocreales*) also appears quite abundant with a prevalence reaching 3%, mainly recorded in the cultivated sites (4% in average) and the lowest value in the woody site (0,5%).

### Diversity and species composition

A finer, manual, bioinformatic analysis on the most abundant and/or significant OTUs was performed to allow resolution at the species level. This was functional to obtaining a clearer and more comprehensive picture of the diversity and of the differences among orchards, grassland, and woodland. Since *Ascomycota* and *Basidiomycota* are the most represented phyla, OTUs belonging to these taxa have been considered only if representing ≥1% of total diversity in at least one sample, or in any case if exclusively retrieved in one sampling site. On the contrary, all OTUs belonging to other, rarer, phyla (i.e. *Zygomycota*, *Glomeromycota*, *Chitridiomycota*) have been considered and analyzed.

**Figure 6:**
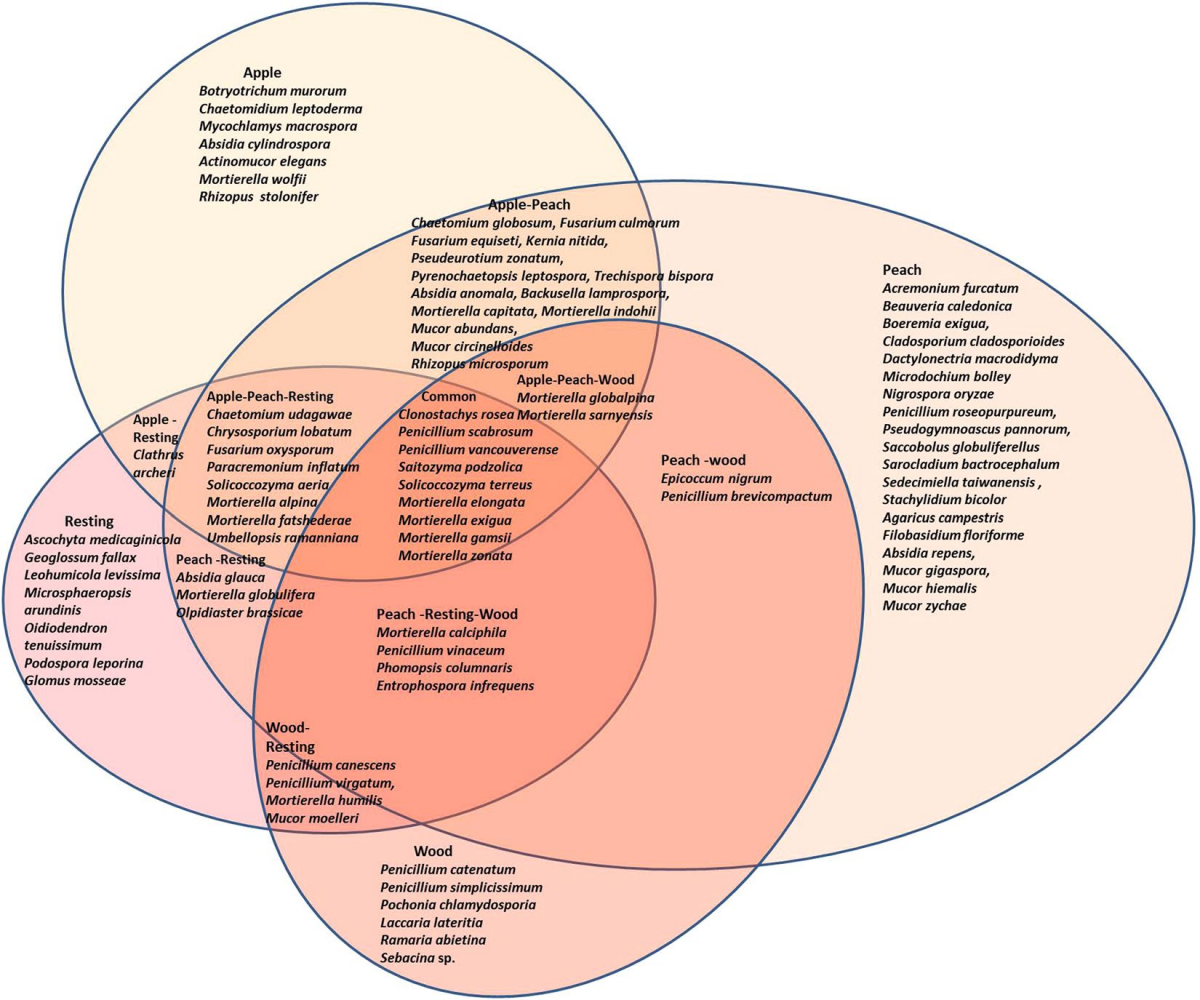
Venn’s diagram that visualizes the general mycobiota, the species that are unique to the sampling sites and those are shared. Species of *Ascomycota* and *Basidiomycota* with an abundance less than 0,5% were not considered.

In each plot, diversity and richness indexes were investigated at the level of OTUs considered as above. The results are reported in Table 3. Soil samples showed similar data especially with respect to the Shannon diversity estimator. Woodland and uncultivated grassland are characterized by the lowest number of observed OTUs and consequently by the lowest Chao1 estimated richness as well as ACE index. Conversely, the area with the highest Chao1 value is peach A (Fig. 6), in accordance with the ACE index.

**Table 3:** Biodiversity and richness estimators calculated at the taxonomy rank of OTUs.

	N	Shannon	Chao1	SE.Chao1	ACE	SE.ACE
appleA	294	4.16	350.67	16.12	369.44	9.69
appleB	312	4.22	367.88	16.81	372.44	9.26
peachA	388	4.42	445.11	16.15	454.96	10.35
peachB	260	4.50	327.86	20.50	324.33	8.82
Resting grassland	222	4.49	238.74	7.32	247.01	7.38
woodland	170	4.16	215.54	16.82	215.66	7.24

Number of OTUs (N), Shannon’s index of biodiversity (Shannon), Chao1 richness estimator (Chao1) and associated standard error (SE.Chao1), Abundance Coverage Estimator (ACE) and corresponding standard error (SE.ACE).

A Venn’s diagram is reported in Fig. 6 to better visualize both the magnitude of the mycobiota and those genera and species that are unique to certain sampling sites and those that are shared. The species found in all the plots are a relatively small group, belonging to the three main phyla (*Ascomycota*, *Basidiomycota* and *Zygomycota*) where the genus *Mortierella* is present with the highest number of species. Peach plot harbors the highest number of exclusive species mainly belonging to *Ascomycota*, followed by the apple and the uncultivated grassland (resting) plots. Most of the basidiomicetous fungi are restricted to woodland plot (e.g. *Laccaria*, *Ramaria*), consistently with their mycorrhizal association with trees.

Supplementary Tables 2–6 show the list of the identified species organized in the context of their taxonomic rank, their relative abundance in the different sites and accession numbers of best hits in public databases.

*Ascomycota* is represented by 77 species, listed in Supplementary Table 2. They appear distributed in all the plots, with a 65% prevalence in orchards and 35% in non-intervened sites. *Penicillium* is the most represented genus and resolves into 13 species, followed by *Acremonium* (6 species) and *Sarocladium* (3 species). The most abundant species are *Penicillium virgatum*, *Penicillium scabrosum*, *Chaetomium udagawae*.

Uncultivated grassland, for its part, is characterized by the dominance and exclusively presence of *Geoglossum fallax* (7%). Together with *Chaetomium udagawae* (see below), *G*. *fallax* is one of the most abundant fungi in the study area. This species belongs to a group of fungi named waxcap which includes genera of grassland fungi that are restricted to soils low in enriching nutrients^41^. Many of these species are typical members of ‘unimproved grassland’, non-intensively managed semi-natural grassland habitats that have not been treated with nitrogen fertiliser^42^. Taxa assigned to this group are considered to be of conservation significance in several European countries. In this study. *G*. *fallax* presence is exclusively associated to the grassland site, an 10 year-abandoned area that can be considered a semi-natural habitat, and its finding is consistent with the literature. This species is considered as a bioindicator of habitat worth to be conserved.

Notable differences, in comparison with other sites, have also been recorded for *Microsphaeropsis arundinis* (4%), *Phomopsis columnaris* (2%), *Ascochyta medicaginicola* (1%). The listed species are often associated to plants as pathogens or endophytic fungi^43^.

The following ascomycetous species result instead exclusive of the woody site: *Oidiodendron tenuissimum* (2%), and *Penicillium catenatum* (1%). Relevant differences are recorded for *Pochonia chlamydosporia* (2,5%) and *Penicillium simplicissimum* (ca. 2%).

The presence of *Oidiodendron* in the woody site is consistent to its ecology. This is a cosmopolitan *genus* whose members can usually be found colonizing different cellulose substrates, i.e. litter, wood pulp, bark, and mosses^44^.

On the whole, samples from the apple orchard A and B appear very similar in the distribution of fungal species. When compared with the other sites, the apple orchards result remarkably different for the presence of *Chaetomium udagawae* (10%), *C*. *globosum* (3%), *C*. *leptoderma* (ca. 1%), *Pseudeurotium zonatum* (ca. 1,5%), and *Mycochlamys macrospora* (ca. 1%). Basing on our knowledge, ecological data on *Chaetomium udagawae* are completely lacking in the literature. In the present study *C*. *udagawae* is the most abundant and its dominance in the area is worth to be investigated in a future research. The species recorded (mainly *C*. *globosum* and *C*. *udagawae*) are dominant in the cultivated areas. The authors assume that their presence is probably due to the high level of soil fertilization (see Supplementary Table 1). Soytong *et al*.^45^, in their review article reported *Chaetomium* species as potent degraders of organic material and often associated to dung^46,47^; in the present study the soil fertilization could have strongly supported these species in cultivated areas.

On the contrary, the soils named “peach A” and “peach B” (referring to two distinct orchards, the former south-east-exposed, and the latter north-west exposed) appear notably different in the distribution of the fungal species (the peach A soil hosts 24% more species than peach B), although this difference is difficult to be explained basing on the site characteristics, e.g. sun exposure. Moreover Peach A and Peach B sites are very similar in the agronomic management and other reasons must be found, e.g. the proximity of rich propagule fungal source areas could explain such data.

The peach A site is characterized mainly by the presence of the following species, exclusive of this site: *Cephalotrichiella penicillata* (3%), and *Pyrenochaeta lycopersici* (ca. 1%). Moreover, the following species are recorded with relevant differences in abundance compared to the other sites: *Penicillium scabrosum* (12%), *Stachylidium bicolor* (3%), *Pseudogymnoascus pannorum* (3%), *Penicillum brevicompactum* (2,5%), *Epicoccum nigrum* (ca. 3%), *Acremonium furcatum* (2,5%), *Cladosporium cladosporioides* (1%).

On the other hand, the site named peach B mainly harbors the following species exclusive of this area: *Beauveria caledonica* (3%) and *Saccobolus globuliferellus* (2%). The latter is a fungus strongly associated to dung and its presence is consistent with the organic fertilization occurring in the orchards^48^.

Supplementary Table 3 lists 28 described species belonging to *Basidiomycota*. The peach A site results twofold richest in basidiomycetous species compared to the other sites. Many species are exclusively retrieved in this site, although with very low abundance, e.g. *Agaricus bisporus*, *Conocybe inopinata*, *Coprinopsis luteocephala*, *Coprinopsis radiate*, *Filobasidium magnum*, *Hannaella oryzae*, *Naganishia diffluens*, *Erythrobasidium hasegawianum*. The only exclusive species with a relative abundance of at least 1% is *Filobasidium floriforme* (ca. 2%) often reported in literature associated to fruits and leaves and frequently used as biocontrol agent against plant diseases^49^.

Other basiomycetous species are rarely recorded in cultivated sites except *Agaricus campestris* which is the most abundant (3,5%), and yeasts mainly represented by *Solicoccozyma terreus*, *S*. *aeria*, *Saitozyma podzolica* (3–4%), species frequently isolated from soil^50^.

The woodland site harbors the exclusive presence of *Laccaria lateritia* (ca. 2%), *Ramaria abietina* (1%), and *Sebacina* highly frequent (5%); their presence can be due to the particular habitat of the woody area. In fact, these genera are reported forming mycorrhizae with trees, and orchid species (*Sebacina* in particular).

For *Zygomycota*, 31 species were recognized (Supplementary Table 4) that mainly belong to the genera *Mortierella* (15 species) and *Mucor* (8 species) and reach the highest prevalence in both samples belonging to the apple orchards and in the peach A site. The uncultivated grassland and woodland sites harbor about half number of species (11 and 10 respectively). The apple sites are characterized by the exclusive presence of *Mortierella exigua*, *Mortierella wolfii*, *Absidia cylindrospora*, and *Actinomucor elegans*.

*Absidia repens, Mucor gigaspora, Mucor hiemalis*, and *Mucor zychae* var. *linnemanniae* are exclusive of peach A.

Only two species, belonging to *Glomeromycota*, have been resolved at the species rank (Supplementary Table 5): *Glomus mossae*, exclusively recorded in the uncultivated grassland field, and *Entrophospora infrequens*.

Finally, *Olpidiaster brassicae* is the only identified *Chytridiomycota* (Supplementary Table 6), mainly in the uncultivated grassland.

Although the present study revealed a huge number of species, many are unidentified mainly in the *Trechisporales* order, or *Chytridiomycota* and *Glomeromycota* phyla, revealing that fungal biodiversity of the upper Andean Colombian agro-environment is still unrevealed.

### Structure of fungal communities in relation to different agro-environmental land use

**Figure 7:**
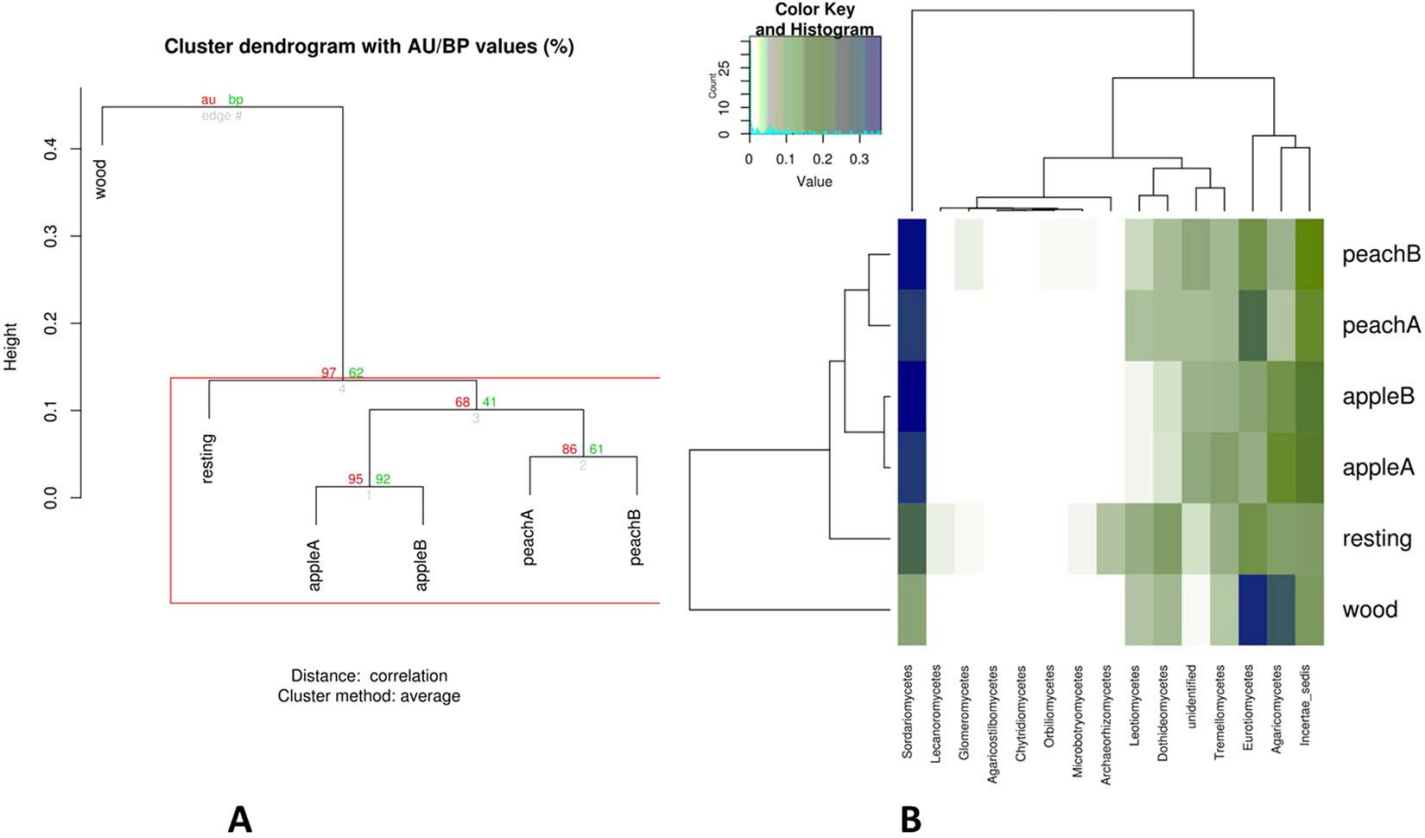
Clustering of samples with respect to their composition in fungal classes. (**A**) Bootstrap-based clustering analysis. (**B**) Heatmap analysis based on the Euclidean distance of classes and on the dendrogram produced by the clustering analysis. AU: approximately unbiased; BP: bootstrap probability.

Samples were compared and clustered with respect to their composition in fungal classes. From the bootstrap-based clustering analysis (Fig. 7A), it clearly emerges that samples are robustly divided into two macro-clusters. The first one includes the woodland area that clusters alone, supported by a highly significant bootstrap value (97%). The second cluster covers the remaining samples. The uncultivated field (resting) is intermediate between orchards (that cluster on the basis of the cultivation type) and woodland. Clustering is mainly dictated by the differential abundance of a limited number of fungal classes, as clearly observable in Fig. 7B: *Sordariomycetes*, *Archaeoryzomycetes*, *Leotiomycetes*, *Dotideomycetes*, *Tremellomycetes*, *Eurotiomycetes*, *Agaricomycetes*. The last two classes, in particular, appear particularly abundant in the woodland sample. This is particularly clear for *Agaricomycetes*, known as strong wood-decayers and ectomycorrhizal symbionts of forest trees, such as pines, oaks, and eucalypts^51,52^. The presence of *Eucalyptus* and *Pinus* species in the woodland site seems to give reason for its presence. *Eurotiomycetes* is a class strongly represented by order, e.g. *Eurotiales*, rich in saprobic species that can find a rich litter to degrade in the woodland. On the other hand, woodland soil presents the lowest richness in *Sordariomycetes*. On the contrary this class is particularly represented in the other plots with abundant coprophilous species (e.g. *Chaetomium globosum*), probably favored by the presence of the organic fertilizer (see Supplementary Table 1) in the orchards and sheep dung in the grassland.

**Figure 8:**
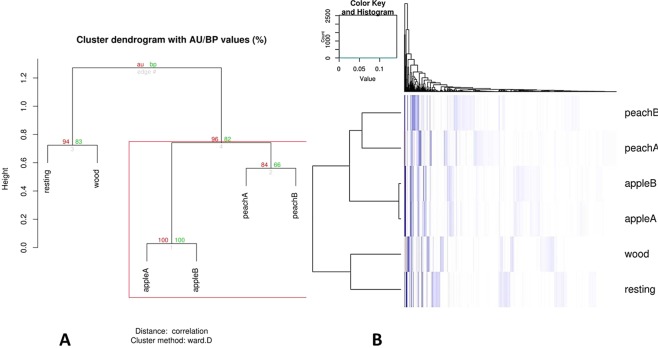
Clustering of samples with respect to their composition in fungal OTUs. (**A**) Bootstrap-based clustering analysis. (**B**) Heatmap analysis based on the Euclidean distance of fungal OTUs and on the dendrogram produced by the clustering analysis. AU: approximately unbiased; BP: bootstrap probability.

Samples were also compared and clustered with respect to their composition in fungal OTUs. From the bootstrap-based clustering analysis (Fig. 8), it clearly emerged that samples were divided into two macro-clusters accordingly to the fungal OTU analysis. The first one included the woodland and the uncultivated area supported by highly significant bootstrap value (94%). The second cluster covered the orchards, showing the two sub-clusters of apple and peach area (Fig. 8A). Differences in land use among the plots (agricultural areas, woodland and semi natural grassland) explain the taxonomic diversity observed in the soil fungi. This result is consistent with that obtained by other authors^53,54^ on diversity of soil or airborne fungi. Moreover, the different management of the cultivated area (peach and apple plots) seems to explain the difference observed in the taxonomic diversity of fungi clearly underlined by the presence of the two sub-clusters (apple and peach plots) in Figs 7 and 8. This result confirms what has been reported by other authors^16,19^ for the soil mycobiota associated with soils subjected to different soil managements.

### Conclusive remarks

Based on the picture emerging from the species composition and from the structure of fungal communities as depicted by the population analysis, some conclusive remarks might be drawn. Both cultivated and not cultivated appear dominated by species belonging to the *Ascomycota* phylum, in agreement with the international literature^11,16,19,55^. Basidiomicetous species are mainly recorded in the woodland area, consistently to the fact that many of them, as saprotrophic or symbiontic (parasitic or mutualistic), show preferences for lignocellulosic material. *Zygomycota* appear homogenously distributed, although with a slightly higher abundance in cultivated areas probably due to their common association to organic fertilization^56^. Among families, *Trichocomaceae* results the most abundant followed by *Mortierellaceae*, and *Nectriaceae*. The finer, manual, bioinformatics analysis on OTUs many taxa were identified at species level: 77 species were identified belonging to *Ascomycota*, 28 to *Basidiomycota*, 31 to *Zygomycota*. Only *Glomus mosseae* and *Entrophospora infrequens* were identified for the *Glomeromycota* phylum; for *Chytridiomycota* only *Olpidiaster brassicae* was identified. The low numbers obtained for *Glomeromycota* species, compared to what expectable from previous studies is likely ascribable to known limits of universal primers targeting the ITS1 barcode^17^. Other gene sequences could be helpful to obtain a major resolution of taxa such as *Glomeromycota* and *Chytridiomycota*.

The uncultivated areas (grassland and woodland) are characterized by the abundant presence of some exclusive species belonging to *Ascomycota*, and *Basidiomycota* (for woodland alone). Particularly important for conservation, is the exclusive presence of *Geoglossum fallax*, the dominant species in the grassland plot; this species is considered as a bioindicator of habitats worth to be conserved.

Many basidiomicetous species, different from those retrieved in the woodland, characterized the peach orchards. This result needs to be deeply investigated with respect to the environmental and management conditions. These cultivated areas result particularly rich in *Chaetomium udagawae* and *C*. *globosum*: this is another interesting aspect that is worth addressing in future studies. From the bootstrap-based clustering analysis, fungal communities split into two main macro-clusters: the woodland-grassland and the orchards. This result seems to indicate a response of microbial community to different use of soil in the agro-environment. The influence of land use and managements on microbial communities were already reported in the literature and our findings confirmed this results also for the particular habitats of upper Colombia.

Retrieved differences in fungal species composition are consistent with the bootstrap-based clustering analysis based on class composition. Considering the study area as a whole, the results obtained seem to draw a taxonomic gradient with the woodland as the most different site compared to cultivated sites and the uncultivated grassland in an intermediate position. Other areas in upper Andean Colombian agro-environments are worth to be investigated in order to confirm the main structure of soil fungal community here reported.

## Materials and Methods

Area of Study and sampling sites. The study area is located in the department of Boyacá (Fig. 1), in Soracá (Colombia), at an altitude of 2900 m a.s.l. (coordinates: 5° 30′ N, 73° W). The climate of the area is classified as “bimodal” with a wet (April-June) and a dry season (July-March). Mean annual temperature is 12 °C, mean precipitation is 900 mm/y, humidity is 75% and winds are almost constant (18 Km/h from South-North). The daily temperature fluctuates meanly between 1 °C and 23 °C.

The sampling site is located in the experimental farm San Isidro Labrador (Fig. 2) belonging to the Fundación Universitaria Juan de Castellanos and located in the Village of Soracá. The site lies on a low hill (almost 7 mt above the level of the plain). Soil samples were collected from three areas differently used: a cultivated area, a woodland area and an abandoned grassland area. In the cultivated area different two fruit trees (apple and peach) are cultivated under agronomic management briefly reported in Supplementary Table 1. The sampling plots are listed below:

- A plot, with an area of 7,454.53 m^2^, South East-exposed, in which peach trees var. “Dorado” have been planted since 2011. Hereafter named “peach A”;
- A second plot, with an area of 6,690.61 m2, North West-oriented, planted with peach trees “Dorado”. Hereafter named “peach B”;
- A plot (4405.95 m2), North West oriented, in which apples (var. “Anna”) are planted. Two different subplots have been sampled named “apple A” and “apple B”.
- A control plot (2500 m2) represented by an uncultivated field, North West-oriented, abandoned for 10 years, dominated by graminaceous plants. Hereafter named “resting grassland” (“resting” in the table and graphs).
- A further control plot, named “woodland” (“wood” in the tables and graphs), represented by a woodland, South East-oriented, planted 10 years ago with alloctonous (*Eucalyptus globulus*, *Pinus* spp., *Acacia* spp.) and autochthonous plants (*Vaccinium meridionale*, *Myrtus communis*, local grass). This area spans 500 m^2^ and is adjacent to the edges of the experimental farm.

### Sample collection

Soil samples were collected during the dry season (January 2016). For soil fungal charge, metabarcoding, and physico-chemical analyses, three soil samples were collected in each plot on the vertices of a triangle with a sampling point placed as far as possible from the others, but at least 10 m away from field edges. Soil samples (500 g each) were aseptically collected at 3 cm depth with a sterile spoon, after removing vegetation cover, debris, and stones, and put in sterile polyethylene bags. Samples were maintained at low temperatures until processed: −20 °C for metabarcoding and +4 °C for fungal counts. For metabarcoding, samples referring to the same plot or subplot were pooled before freezing.

### Soil chemical analyses

Chemical properties of soils were determined by Labanalysis, Casanova Lonati (Pavia, Italy), according to the Italian standard protocols (DM 13/09/99). The following parameters were evaluated: pH, organic matter, total nitrogen (N_TOT_), organic carbon (C_ORG_), C/N ratio, plant-available phosphorous (P), calcium (Ca), magnesium (Mg), potassium (K), soil composition in sand, silt, and clay.

### DNA extraction, ITS1 amplification and Illumina sequencing

Total DNA was extracted starting from 350 mg of each sample with the NucleoSpin Soil kit (Macherey-Nagel, Düren, Germany) according the manufacturer’s specifications and quantified using a Qubit fluorometer (ThermoFisher Scientific, Waltham, MA). For amplicon production, the ribosomal ITS1 region (Internal Transcribed Region 1) was targeted, by using primers BITS and B58S3^57^ linked to Illumina adapters. PCR was performed in a 50-µl volume containing 5 to 10 ng template DNA, 1x HiFi HotStart Ready Mix (Kapa Biosystems, Wilmington, MA), 0.5 µM of each primer. The cycling program, performed on a MJ Mini thermal cycler (Promega corp., Madison, WI), included an initial denaturation (95 °C for 3 min), followed by 25 cycles at 94 °C for 30 s, 55 or 60 °C C for 30 s, 72 °C for 30 s, and final extension (72 °C for 5 min). Clean-up of amplicons was performed using Agencourt AMPure XP SPRI magnetic beads (ThermoFisher Scientific). Illumina sequencing libraries were finally constructed through the link of indexes (Nextera XT Index Kit, Illumina, San Diego, CA), quantified using a Qubit 2.0 Fluorometer (ThermoFisher Scientific), normalized and pooled. Libraries were subjected to paired-end sequencing (2 × 250 bp, nano format) on an Illumina MiSeq sequencer at BMR Genomics (Padova, Italy). The sequencing data were deposited in The European Nucleotide Archive (ENA) under the Accession Number PRJEB32659.

### Data analysis: definition of operational taxonomic units (OTUs) and community analyses

Data analysis was performed using the pipeline PIPITS^40^. Raw data were demultiplexed and barcodes and primers were trimmed off. Quality filters were applied, including a length threshold (100 bp) and removal of singletons. High-quality reads were clustered into operational taxonomic units (OTUs) at 97% similarity using VSEARCH^58^ and chimaeras excluded using UCHIME^59^. OTUs were finally annotated using the UNITE fungal ITS reference data set within RDP classifier (http://rdp.cme.msu.edu) and the Worcup ITS reference as training dataset.

Species were assigned on selected OTUs against mycobank (mycobank.org), RDP (https://rdp.cme.msu.edu/) and using the specialized fungal pages within GenBank. Finally, relative abundances of microbial taxa in each sample were calculated and compared.

α-diversity matrices were assessed by computing Chao 1, Abundance Coverage Estimator (ACE) and Shannon indexes at the level of OTUs using the R library Vegan^60^. Diversity in composition among samples (β-diversity) was compared, at all taxonomic ranks, through: (i) bootstrap-based clustering analysis, using the R function pvclust^61^; (ii) double clustering analysis, through the R function heatmap.2 of the R library G plots^62^, setting the pvclust output as sample dendrogram; (iii) Principal Coordinates Analysis (PCoA) analysis based on Bray-Curtis dissimilarity matrix, performed using the R library Vegan^60^.

### Evaluation of total fungal count

After collection, soil samples were processed within 15 days, using the Dilution Plate Technique^63^ to detect, quantitatively, cultivable microfungi. According to previous studies^64,65^, 10 g of each soil sample was suspended 1:10 (w/v) in sterile water + agar (0.15%) and shaken for 20 min. Suspensions were further diluted 1:10 and 1 ml aliquots of the 10^−4^ suspension were homogeneously distributed with a sterile blent rod onto 16 cm diameter Petri dishes containing MEA medium (Malt Extract Agar – CBS: 20 g malt extract powdered, 1 g peptone, 20 g glucose, 15 g agar, 1000 ml distilled water) and antibiotics (streptomycin 20 ppm, chloramphenicol 100 ppm, penicillin G 30 ppm). Five replicates of each sample were prepared, and incubated at 25 °C in the dark. Inoculated plates were observed continuously during 2 weeks by means of a stereomicroscope, and the number of developed colonies was expressed as CFU (Colonies Forming Units) per gram of soil (dry weight). Soil dry weight was measured by an infrared moisture balance (AMB Adam Equipment, UK).

## Supplementary information

Legends of supplementary tables

Data set 1

Data set 2

Data set 3

Data set 4

Data set 5

Data set 6
