# Supplementary material for: A meta-barcoding analysis of soil mycobiota of the upper Andean Colombian agro-environment
Source: Sci Rep. 2019 Jul 12;9:10085. doi: 10.1038/s41598-019-46485-1 (PMC6625999; doi:10.1038/s41598-019-46485-1)
Supplement: Supplementary file 1 — Legends of supplementary tables [file 41598_2019_46485_MOESM1_ESM.pdf]

A meta-barcoding analysis of soil mycobiota of the upper Andean Colombian agro-environment

Angela Landinez-Torres [a, b§], Simona Panelli [c§], Anna Maria Picco [a], Francesco Comandatore [c], Solveig Tosi [a*], Enrica Capelli [d, e]

[a] Mycology Laboratory, Department of Earth and Environmental Sciences, University of Pavia, Via Ferrata 7, 27100, Pavia, Italy

[b] Facultad de Ciencias Agrarias y Ambientales, Fundación Universitaria Juan de Castellanos. Carrera 11 No. 11 - 44 Tunja, Boyacá – Colombia

[c] Pediatric Clinical Research Center Romeo ed Enrica Invernizzi, University of Milan, Via G.B. Grassi 74, 20157, Milan, Italy

[d] Genetic Analysis Laboratory, Department of Earth and Environmental Sciences, University of Pavia, Via Ferrata 7, 27100, Pavia, Italy

[e] Centre for Health Technologies (C.H.T.), University of Pavia, Via Ferrata 1, 27100, Pavia, Italy

§ Joint first authors

* Corresponding author

Email address: solveig.tosi@unipv.it

Supplementary information
Legends for supplementary tables

Supplementary table 1. Agronomic management of orchards

Supplementary table 2. *Ascomycota* OTU analysis and identification: Fungal species identified in the different sampling sites, ordered following their taxonomy. Abundance (%) per site is shown. Identifications have been performed against the GenBank, Mycobank and RDP classifier databases.

Supplementary table 3. *Basidiomycota* OTU analysis and identification: Fungal species identified in the different sampling sites, ordered following their taxonomy. Abundance (%) per site is shown. Identifications have been performed against the GenBank, Mycobank and RDP classifier databases.

Supplementary table 4. *Zygomycota* OTU analysis and identification: Fungal species identified in the different sampling sites, ordered following their taxonomy. Abundance (%) per site is shown. Identifications have been performed against the GenBank, Mycobank and RDP classifier databases.

Supplementary Table 5. *Glomeromycota* OTU analysis and identification: Fungal species identified in the different sampling sites, ordered following their taxonomy. Abundance (%) per site is shown. Identifications have been performed against the GenBank, Mycobank and RDP classifier databases.

Supplementary Table 6. *Chytridiomycota* OTU analysis and identification: Fungal species identified in the different sampling sites, ordered following their taxonomy. Abundance (%) per site is shown. Identifications have been performed against the GenBank, Mycobank and RDP classifier databases.
